# Prolonged development of tonotopic tuning in human auditory cortex

**DOI:** 10.64898/2026.04.20.719686

**Published:** 2026-10-14

**Authors:** Oyindamola Ogunlade, Jesse Gomez

## Abstract

Audition is a fundamental sense, underlying critical human behaviors such as communication and recognition. Despite its importance, how the tuning and organization of receptive fields mature from childhood to adulthood in auditory cortex has not been directly measured. Through a gamified neuroimaging approach using functional MRI, we model population receptive field (pRF) tuning for frequency across human auditory cortex in both children and adults. In the same participants, we behaviorally quantify detection thresholds for different frequencies embedded in noise to understand how the functional development of human auditory cortex drives behavior. We find that while the tonotopic organization of pRFs is qualitatively present in early childhood, there is a protracted increase in the representation of low frequencies in tonotopic maps of auditory cortex. This maturation of pRF tuning appears to drive basic auditory behaviors, relating to tone detection thresholds across participants. We also observe protracted development in secondary auditory regions, offering evidence for an anatomically-predictable tonotopic map posterior to Heschl’s Gyrus. These data provide a new avenue for studying the development of audition in the human brain and lay important groundwork for understanding atypical development in auditory processing disorders.

